# Machine learning-based model for predicting the esophagogastric variceal bleeding risk in liver cirrhosis patients

**DOI:** 10.1186/s13000-023-01293-0

**Published:** 2023-02-23

**Authors:** Yixin Hou, Hao Yu, Qun Zhang, Yuying Yang, Xiaoli Liu, Xianbo Wang, Yuyong Jiang

**Affiliations:** 1 Center of Integrative Medicine, Beijing Ditan Hospital, Capital Medical University, No. 8 Jingshun East Road, Beijing, 100051 China; 2 Institute of Liver Diseases, Shuguang Hospital Affiliated to Shanghai University of Traditional Chinese Medicine, Shanghai, China

**Keywords:** ALT, Artificial neural network, Ascites, Gastroesophageal varices, GG, Hematocrit, Neutrophil-lymphocyte ratio, North italian endoscopic club analysis, Red blood cell count, Risk analysis

## Abstract

**Background:**

Liver cirrhosis patients are at risk for esophagogastric variceal bleeding (EGVB). Herein, we aimed to estimate the EGVB risk in patients with liver cirrhosis using an artificial neural network (ANN).

**Methods:**

We included 999 liver cirrhosis patients hospitalized at the Beijing Ditan Hospital, Capital Medical University in the training cohort and 101 patients from Shuguang Hospital in the validation cohort. The factors independently affecting EGVB occurrence were determined via univariate analysis and used to develop an ANN model.

**Results:**

The 1-year cumulative EGVB incidence rates were 11.9 and 11.9% in the training and validation groups, respectively. A total of 12 independent risk factors, including gender, drinking and smoking history, decompensation, ascites, location and size of varices, alanine aminotransferase (ALT), γ-glutamyl transferase (GGT), hematocrit (HCT) and neutrophil-lymphocyte ratio (NLR) levels as well as red blood cell (RBC) count were evaluated and used to establish the ANN model, which estimated the 1-year EGVB risk.

The ANN model had an area under the curve (AUC) of 0.959, which was significantly higher than the AUC for the North Italian Endoscopic Club (NIEC) (0.669) and revised North Italian Endoscopic Club (Rev-NIEC) indices (0.725) (all *P* <  0.001). Decision curve analyses revealed improved net benefits of the ANN compared to the NIEC and Rev-NIEC indices.

**Conclusions:**

The ANN model accurately predicted the 1-year risk for EGVB in liver cirrhosis patients and might be used as a basis for risk-based EGVB surveillance strategies.

**Supplementary Information:**

The online version contains supplementary material available at 10.1186/s13000-023-01293-0.

## Background

Esophagogastric variceal bleeding (EGVB) is a major complication in liver cirrhosis patients, which has a high mortality rate worldwide. Gastroesophageal varices (GEV) are present in about 50% of individuals diagnosed with cirrhosis of the liver. New varices occur at a rate of 3–12% each year, and preexisting varices convert into large varices in 8–12% of patients with liver cirrhosis per year [1]. Combined treatments with non-selective β-blocker therapy, vasoactive drugs, endoscopic therapy, and interventional treatments are effective methods for preventing and controlling esophageal variceal bleeding, which are also recommended for patients with acute variceal bleeding [2, 3]. Despite the marked advances in the management of portal hypertension and EGVB in recent years, nearly 12% of patients experience the first bleeding each year, and over 20% experience re-bleeding within 6 weeks [4, 5]. Therefore, it is important to assess the presence of esophageal varices and therefore the probability of an increased bleeding risk.

A general consensus suggested using risk stratification scores in patients with GEV, which could help assess the risk of bleeding, prevent bleeding, and reduce the mortality of patients [6]. The hepatic venous pressure gradient is an important for stratifying liver cirrhosis patients, which can predict hypertension-related portal circulation complications [7–9]. Nevertheless, the hepatic venous pressure gradient is not available in routine clinical practice because of its invasive nature. A number of markers of fibrosis have been used as predictors to evaluate the risk of the first variceal bleed or indeed re-bleeding in patients with GEV. Because portal hypertension is caused by raised intrahepatic blood vessel resistance and the associated fibrosis and cirrhotic nodules [10–14]. However, there were inconsistent results owing to the heterogeneity of studies with respect to etiology, treatments, prophylactic therapy, and cut-off values.

The combination of endoscopic parameters and clinical indicators is considered an appropriate method to provide an assessment of the EGVB risk [11]. Currently, the most widely used indices for stratifying high-risk patients are the North Italian Endoscopic Club (NIEC) and revised North Italian Endoscopic Club (Rev-NIEC) indices. Both indices are a combination of the Child–Pugh classification and endoscopic parameters, including the size of varices and red wale markings (RWM) [15, 16].

Artificial neural networks (ANN) are mathematical models governed by the biological nervous system similarity to information processing in the central nervous system [17]. There use in diagnosis have the advantages of incorporating complete statistical analyses of numerous complicated relationships of disease [18]. For liver related diseases, ANN models were developed for the diagnosis of cirrhosis in hepatitis B hepatocellular carcinoma (HCC) patients [19] and their mortality [20], and for the prediction of severe liver failure after hemihepatectomy in HCC patients [21]. In addition ANN models were also used for predicting the likelihood of fatty liver disease [22] in addition to the noninvasive diagnosis of biliary atresia [23].

The purpose of the present study was to use for the first time the ANN method to develop an early-stage warning model that could predict EGVB in patients with liver cirrhosis. It could also be used to make comparisons between the results of NIEC and the Rev-NIEC indices.

## Methods

### Patients

Data from 1928 consecutive patients with liver cirrhosis admitted to our hospital between February 2008 and February 2017 were screened retrospectively. The inclusion criteria were: (1) age ≥ 18 and ≤ 75 years; (2) diagnosis of cirrhosis (based on clinical manifestations and imaging as well as blood test or liver biopsy results); and (3) presence of GEV confirmed through an endoscopic examination but without a history of variceal hemorrhages. Exclusion criteria were: (1) age < 18 or > 75 years; (2) complications of liver cancer or other space-occupying lesions; (3) regular use of propranolol or proton pump inhibitors; (4) history of splenectomy, endoscopic treatments, or transjugular intrahepatic portosystemic shunting before or after inclusion in the study; (5) complications with other conditions that may cause bleeding, such as ulcers and coagulation disorders; and (6) follow-up of less than 1 year or missing data. In addition, following the same inclusion/exclusion criteria, patients were selected from Shuguang Hospital affiliated to the Shanghai University of Traditional Chinese Medicine from October 2015 to March 2018, who formed a separate validation cohort.

The present study was approved by the Ethics Committee of our hospital (approval number: 2020–043-02), because of its retrospective nature and written informed consent was obtained from all participants.

### Data collection

The patients’ baseline clinical characteristics and laboratory values were collected at the first endotherapy for variceal bleeding or the first gastroscopy without variceal bleeding, including general demographic characteristics (age and sex), medical history, blood routine examination findings, complications (ascites, bacterial infection, and hepatic encephalopathy), routine laboratory parameters (aspartate aminotransferase, alanine aminotransferase [ALT], total bilirubin, γ-glutamyl transferase [GGT], alkaline phosphatase level, and albumin concentrations, white blood cell, red blood cell [RBC] and platelet counts, the neutrophil-lymphocyte ratio [NLR], hematocrit [HCT], potassium, sodium, blood urine nitrogen, creatinine and glucose concentrations, prothrombin time, international normalized ratio, HBV DNA level), endoscopic parameters (size of varices and RWM), and ultrasonography findings (portal vein diameter and spleen thickness). The Child–Pugh classification and model for end-stage liver disease (MELD) scores were determined to evaluate the liver function status of each patient [24, 25]. All these variables were included in the least absolute shrinkage and selection operator Cox regression analysis to filter the candidate variables for the model. The NIEC and Rev-NIEC indices were calculated according to previously published criteria [15, 16]. All prognostic scores and definitions were applied at baseline.

### Clinical definition and follow-ups

Construction of an ANN: ANN represent complex interconnected processing units (neurons) linked to weighted connections, with inputs, output, and hidden layers [26–29]. ANN incorporate self-learning, self-adapting processes with inference. ANN after ‘learning’ from various inputs are capable of connecting any input to a corresponding output. An input is propagated from the first layer of neurons through each upper layer and an output is produced, together with a process that is self-adapting. If there is a discrepancy between the 2 outputs, an error signal is generated. During learning, the errors between the merits of the generated and desired outputs is decreased until the minimum is achieved. Subsequently, an inference process is conducted, when the output (prognosis) can be generated from the input data based on knowledge accumulated during the training process. ANN can therefore accurately predict data sets [26–29].

In the present study, variables which were significantly associated with EGVB in the patients with cirrhosis were used to construct ANN using Mathematica ver. 11.1.1 (Microsoft). The learning process of each ANN was conducted using back propagation (BP) by assess any errors between values of the generated and expected outputs. The weight of the interneuron connections was adjusted to minimize the overall potential network errors. Learning (training) ceased when the total square errors were at a minimum compared to the cross-validation dataset. Finally, the output form provided data on the potential risk for each patient with liver cirrhosis to develop EGVB within 1 year.

### Statistical analyses

Data are given as medians (ranges) or as n (%). Differences among continuous or categorical variables were assessed using a Student’s *t*-test or Mann–Whitney, chi-squared or Fisher’s exact tests. Hazard ratios and 95% confidence intervals (CIs) along with the corresponding *P*-values are given.

Discrimination performance was evaluated using receiver operating characteristic curves with the area under the receiver operating characteristic curves (AUROC) computed to generate Harrell’s concordance index (C-index). We also compared the ANN model with the well-established NIEC and Rev-NIEC index models in relation to the operating characteristic curves. These scores were calculated according to a previously published scoring formula. A calibration plot was used to graphically assess the agreement between the probability of non-development of EGVB within 1 year, as predicted by the model in comparison with the observed probability. Analysis of decisions curves was employed to make comparisons between clinical net benefits of the new and previous models. For all tests, *P*-values < 0.05 were considered to denote significant differences. Statistical analyses were computed using SPSS ver. 22 (IBM, USA) and R version 3.3.2 (R Development Core Team, 2010).

## Results

### Baseline characteristics of participants

**Fig. 1:**
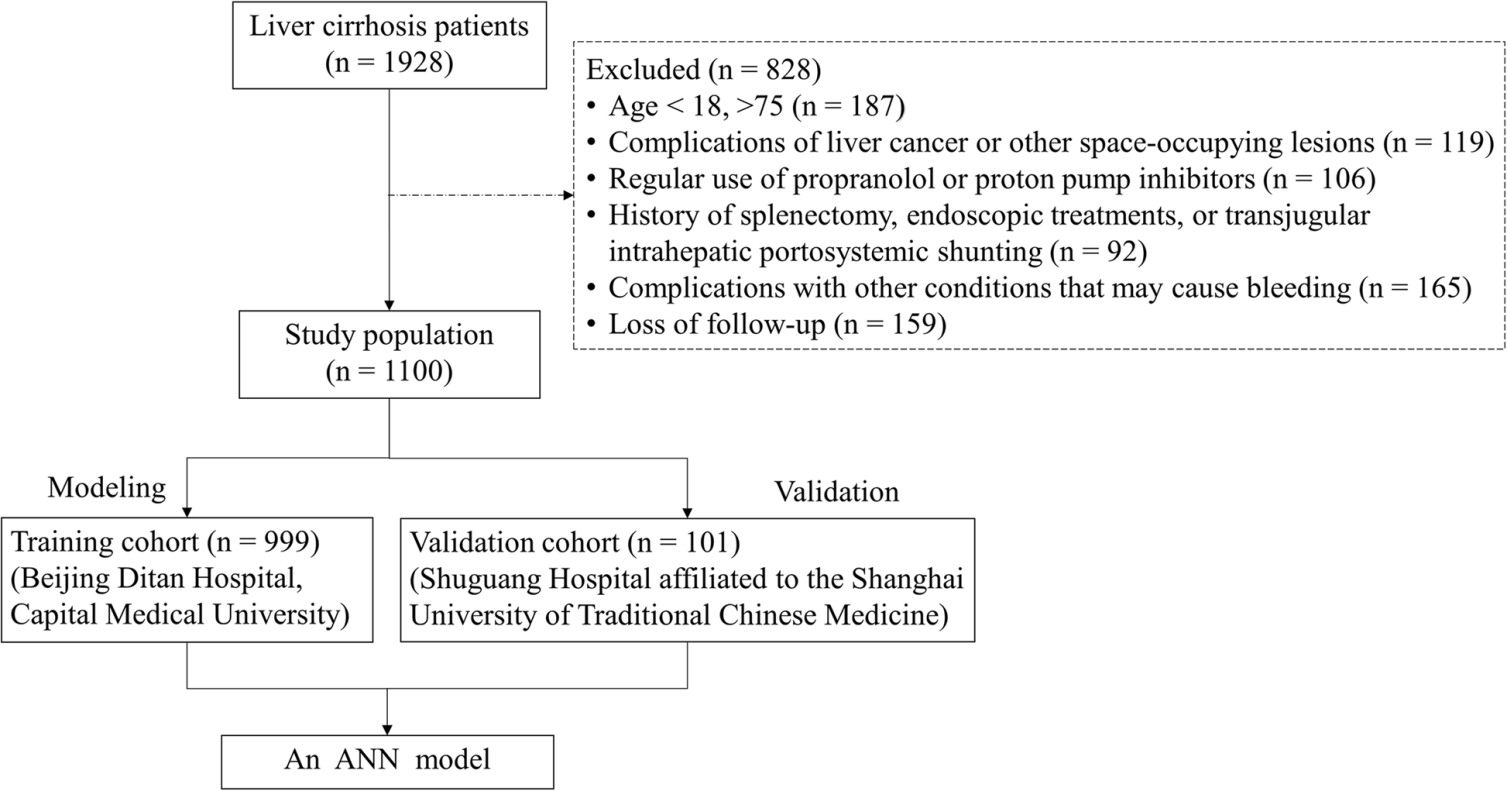
Flow chart

In total, 999 patients comprised the training and 101 the validation cohorts (Fig. 1). Baseline data of the enrolled individuals are listed in Supplementary Table 1 [see Supplementary materials]. A total of 119 (11.9%) and 12 (11.9%) patients in the validation and derivation cohorts respectively, had their first EGVB occurrence during the 1-year follow-up. In the derivation cohort, the median age was 53.0 (interquartile range, 45.0–60.0 years), and 680 (68.1%) of them were males. The most common complication was ascites (56.0%), followed by bacterial infection (19.2%) and hepatic encephalopathy (4.2%). Endoscopy showed that the proportions of small, medium, and large varices were 49.1, 26.2, and 24.6%, respectively. RWM was detected in 30.7% of patients. Most were classified under Child–Pugh grade B (49.0%), followed by grade A (34.6%) and grade C (16.3%), with a median MELD score of 10.0 (IQR, 8.0–13.0). It is noteworthy that the derivation cohort had higher rates of bacterial infections (*P* <  0.05).

### Construction of the ANN model

In a cox univariate regression analysis (Table 1), we found that gender, drinking and smoking history, decompensation, ascites, location and size of varices, ALT, GGT, HCT and NLR levels as well as RBC count were significantly associated with EGVB occurrence in the training group.

**Table 1:** Factors associated with prediction of EGVB

Variables	Univariate analysis		***P-***value^†^
	β	HR (95% CI)	
**Age (years)**	−0.011	0.989 (0.968–1.010)	0.306
**Male gender**	0.618	1.855 (1.051–3.272)	0.033
**Liver disease etiology**			
**Smoking**	0.573	1.774 (1.121–2.807)	0.014
**Drinking**	0.900	2.461 (1.535–3.944)	< 0.001
**Decompensation**	0.998	2.713 (1.429–5.152)	0.002
**Ascites**	0.897	2.452 (1.440–4.176)	0.001
**Hepatic encephalopathy**	0.327	1.387 (0.506–3.800)	0.525
**Bacterial infection**	−0.100	0.905 (0.497–1.648)	0.744
**Location of varices at index gastroscopy**			
**Gastric varices only**	Reference		
**Esophageal varices only**	−0.714	0.489 (0.067–3.602)	0.483
**Esophageal and gastric**	0.910	2.485 (1.542–4.004)	< 0.001
**Size of varices**			
**Small**	Reference		
**Medium**	1.152	3.165 (1.629–6.151)	0.001
**Large**	1.699	5.469 (2.950–10.139)	< 0.001
**Red wale marks**	1.469	4.346 (2.691–7.017)	< 0.001
**Laboratory data**			
**ALT (U/L)**	−0.004	0.996 (0.992–1.000)	0.005
**AST (U/L)**	−0.003	0.997 (0.993–1.001)	0.093
**TBIL (**μmol**/L)**	−0.002	0.998 (0.994–1.003)	0.487
**GGT (U/L)**	0.001	1.001 (1.000–1.002)	0.006
**ALP (U/L)**	−0.001	0.999 (0.995–1.002)	0.548
**ALB (g/L)**	−0.034	0.967 (0.929–1.005)	0.091
**WBC (× 10** ^**9**^ **/L)**	0.004	1.004 (0.897–1.125)	0.938
**RBC (× 10** ^**12**^ **/L)**	−0.425	0.654 (0.477–0.898)	0.009
**PLT (× 10** ^**9**^ **/L)**	−0.002	0.998 (0.992–1.003)	0.414
**NLR**	0.082	1.086 (1.018–1.158)	0.013
**HCT (%)**	−0.053	0.948 (0.919–0.978)	0.001
**K (mmol/L)**	−0.284	0.752 (0.441–1.283)	0.296
**NA (mmol/L)**	−0.006	0.994 (0.936–1.056)	0.848
**BUN (mmol/L)**	−0.072	0.930 (0.824–1.050)	0.243
**CREA (μmol/L)**	−0.005	0.995 (0.983–1.007)	0.381
**GLU (mmol/L)**	−0.091	0.913 (0.814–1.024)	0.119
**PT (s)**	0.014	1.014 (0.939–1.095)	0.721
**PTA (%)**	−0.009	0.991 (0.977–1.004)	0.172
**Spleen thickness (mm)**	0.046	1.048 (1.027–1.068)	< 0.001
**Portal vein diameter (mm)**	0.113	1.120 (0.987–1.270)	0.079
**Child-Pugh grade‡**			
**A**	Reference		
**B**	−0.190	0.827 (0.500–1.369)	0.827
**C**	−0.085	0.919 (0.467–1.807)	0.919
**MELD** ^a^	−0.018	0.982 (0.935–1.032)	0.481

^†^Comparison results between the derivation and validation cohorts

^a^Child–Pugh grade and MELD score were not included in the least absolute shrinkage and selection operator regression analyses

*Abbreviations*: *ALB* Albumin; *ALP* Alkaline phosphatase, *ALT* Alanine aminotransferase, *AST* Aspartate aminotransferase, *BUN* Blood urine nitrogen, *CI* Confidence interval, *CREA* Creatinine, *EGVB* Esophagogastric variceal bleeding; *GGT* γ-glutamyl transferase, *GLU* Glucose, *HBV* Hepatitis B virus, *HCT* hEmatocrit, *HCV* Hepatitis C virus, *HR* Hazard ratio, *K* Potassium, *MELD* Model for end-stage liver disease, *NA* Sodium, *NLR* Neutrophil-lymphocyte ratio, *PLT* Platelet, *PT* Prothrombin time, *PTA* Prothrombin time activity, *RBC* Red blood cell, *TBIL* Total bilirubin, *WBC* White blood cell

**Fig. 2:**
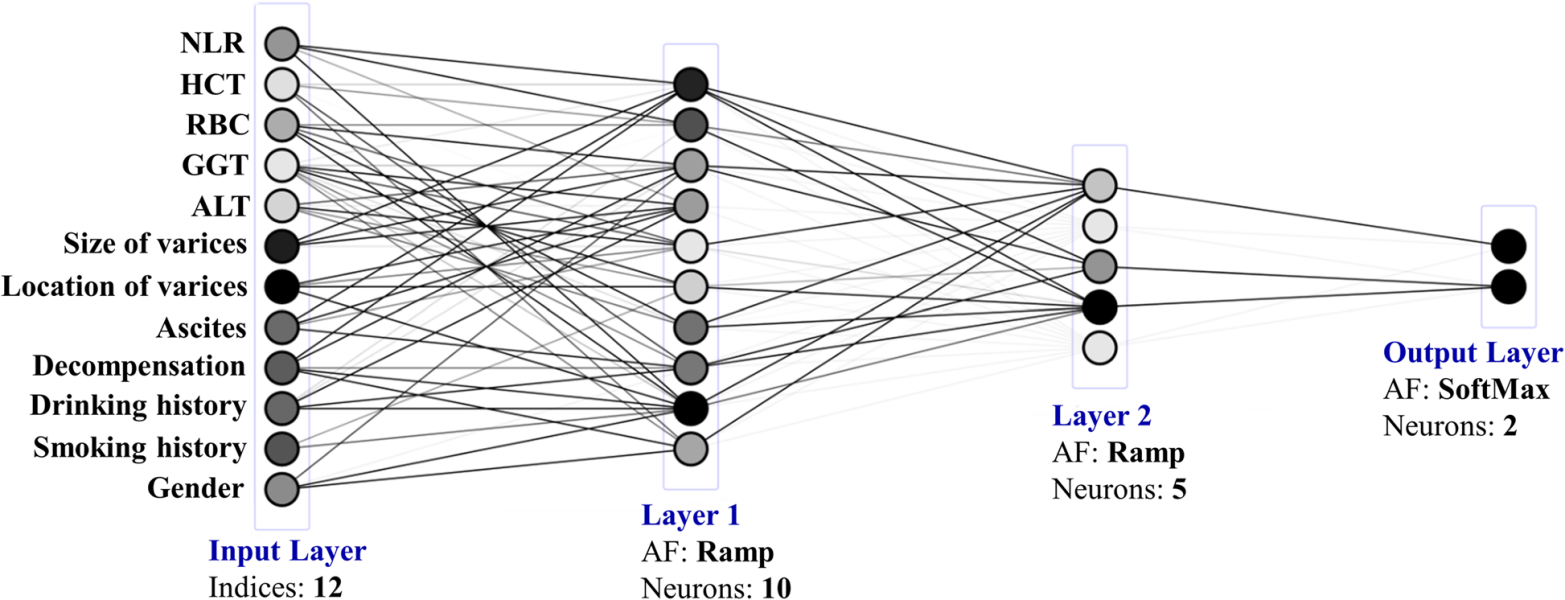
Artificial neural network model page design according to different conditions of patients

The ANN model for the development of EGVB within 1 year in the patients with liver cirrhosis is shown in Fig. 2. Multilayer perceptron is a regular ANN structure, comprised of components including input, hidden and output layers [12]. Clinical and biochemical parameters are included in the input layer (indicated with gray shades in Table 1) with the output layer including a corresponding prognosis outcome. To improve multilayer perceptron performance, 2 hidden layers after much debugging and testing were added.

### Application for risk stratification of the ANN model

All patients were divided into two strata, corresponding to lower and upper quartile scores of the ANN model, namely Stratum 1, low risk and Stratum 2, high risk. With Stratum 1 as the reference, the HR for Stratum 2 was 0.8 (95% CI = 29.11–86.82) (*P* <  0.001) in the training cohort. The ANN model clearly distinguished patients according to various risk factors, regardless of the cohort. The negative and positive prediction values are listed in Table 2.

**Table 2:** Positive and negative predictive values of the ANN

Cohort	Models	1-year risk of EGVB	
		Positive % (95% CI)	Negative % (95% CI)
Training	ANN (low)	26.2 (25.0–27.4)	98.7 (95.2–99.7)
	ANN (high)	54.7 (48.6–60.7)	91.6 (89.4–93.4)
Validation	ANN (low)	20.9 (19.6–22.2)	100 (−)
	ANN (high)	41.5 (32.8–50.8)	91.9 (88.6–94.3)

*Abbreviations*: *ANN* Artificial neural networks, *CI* Confidence interval, *EGVB* Esophagogastric variceal bleeding

**Fig. 3:**
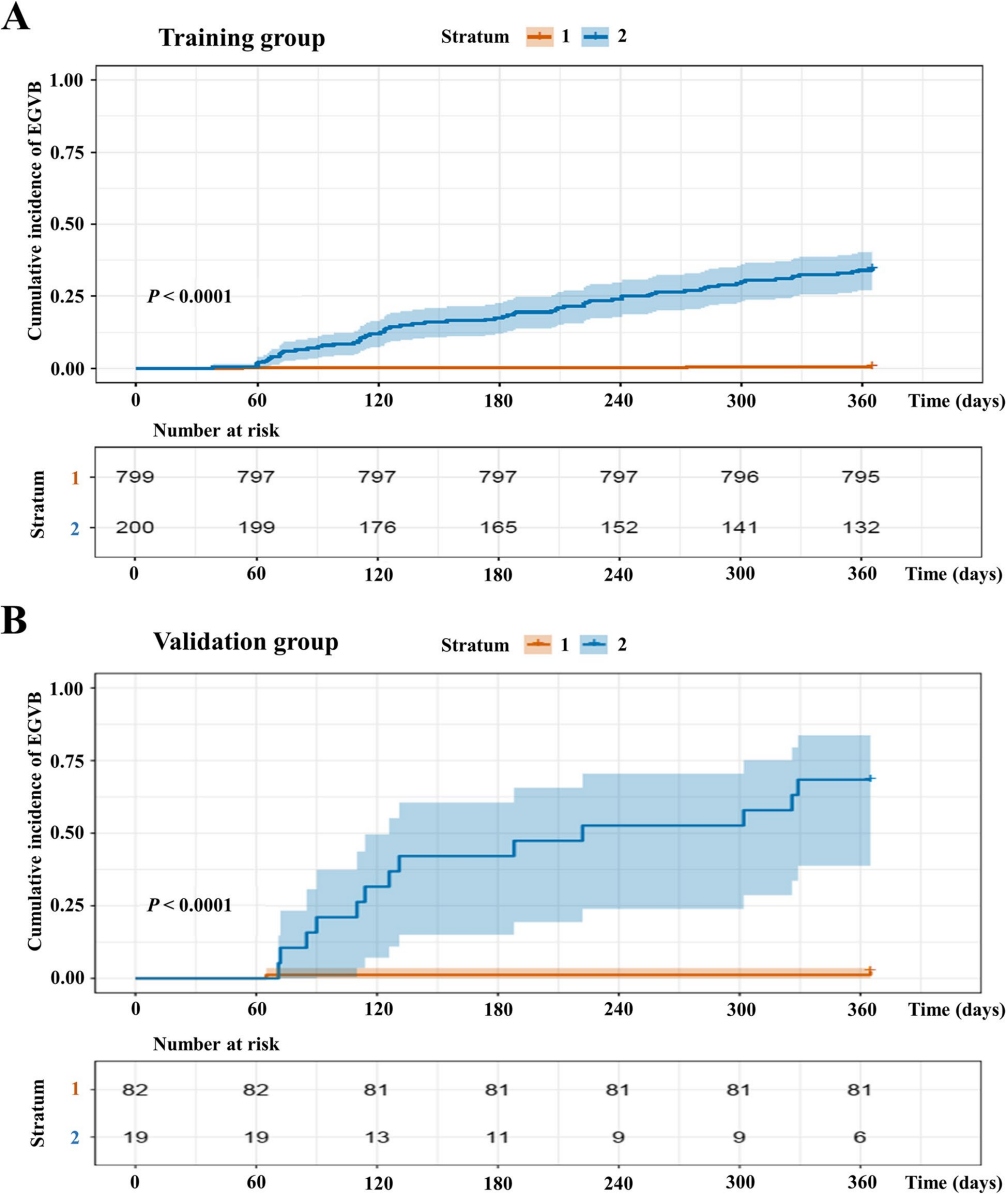
Kaplan–Meier incidence of EGVB in the training (**A**) and validation (**B**) cohorts according to the ANN model division into low (Stratum 1) and high (Stratum 2) risk layers

In the training cohort, the predicted cumulative EGVB incidence coincided with the observed Kaplan–Meier incidence for both the low-risk and high-risk groups (Fig. 3A) and in the validation cohort, the plots revealed an excellent correlation between the observed and predicted cumulative incidence rates (Fig. 3B).

### ANN model calibration and discrimination

In the training cohort, the AUROC of the ANN model for EGVB occurrence was 0.959 (95% CI: 0.945–0.973), and the C-index was 0.956 (95% CI: 0.728–0.972), whereas in the validation cohort the data were 0.945 (0.877–0.987) and 0.936 (0.753–0.965), which was significantly superior to the values of the NIEC and Rev-NIEC indices models (*P* <  0.001) (Table 3).

**Table 3:** Comparison of the performance and discriminative ability between the current and other models

Cohort	Models	1-year risk of EGVB	
		AUROC (95% CI)	C-index (95% CI)
Training	ANN	0.959 (0.945–0.973)	0.956 (0.728–0.972)
	NIEC	0.669 (0.605–0.731)	0.717 (0.646–0.735)
	Rev-NIEC	0.725 (0.669–0.780)	0.681 (0.636–0.726)
Validation	ANN	0.945 (0.877–0.987)	0.936 (0.753–0.965)
	NIEC	0.743 (0.600–0.887)	0.707 (0.643–0.772)
	Rev-NIEC	0.797 (0.667–0.927)	0.701 (0.631–0.771)

*Abbreviations*: *ANN* Artificial neural networks, *AUROC* Area under the receiver operating characteristic curves, *CI* Confidence interval, *C-index* Concordance index, *EGVB* Esophagogastric variceal bleeding, *NIEC* North Italian Endoscopic Club, *Rev-NIEC* Revised North Italian Endoscopic Club

Calibration curves were plotted, which showed good agreement between the ANN model-predicted probability of non-development of EGVB and observed probability within 1 year in the training (Supplementary Fig. 1A) and validation cohorts (Supplementary Fig. 1B) [see Supplementary materials].

## Discussion

Since EGVB prevention is the primary goal of GEV patient management [30], the current major international guidelines recommend EGVB surveillance for cirrhosis using biannual abdominal ultrasonography, regardless of individual risks. However, several decades ago, since researchers noticed the importance of bleeding risk assessment in the development of prevention strategies several models for this purpose were established [15, 16, 31]. Several studies have reported a non-uniform EGVB risk, therefore, a “one-size-fits-all” approach is very likely to underestimate or overestimate the EGVB risk per patient. Utilization rates can be improved using strategies that are risk-stratified, because resources then concentrate on patients at the highest-risk rather than being disseminated between all liver cirrhosis patients. Among them, the NIEC index is the most widely used tool, and the risk stratification based on this index is still an important basis for primary prevention strategies [15, 16, 30].

**Fig. 4:**
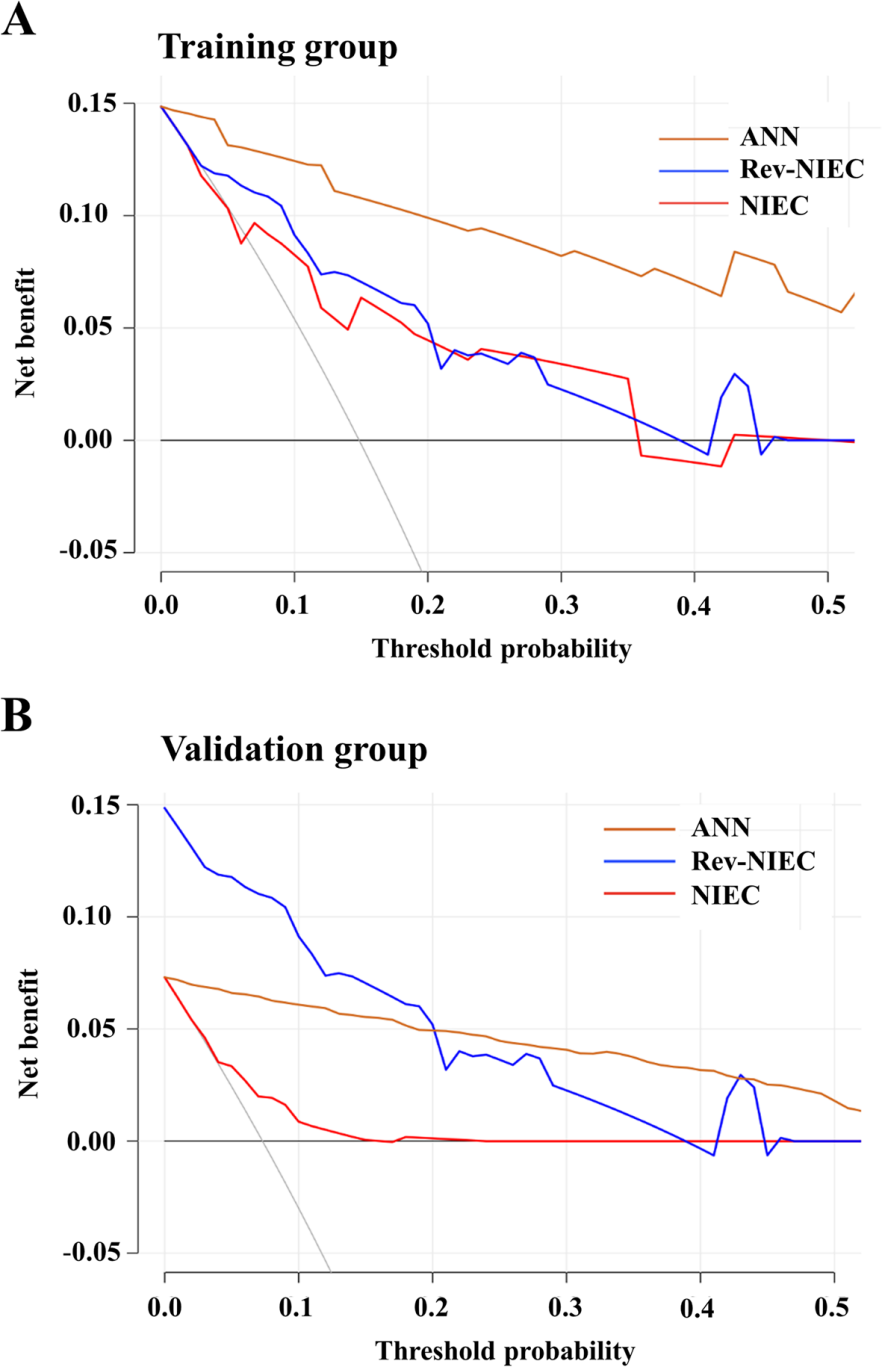
Decision curve analyses for predicting the incidence of gastroesophageal variceal bleeding in the training (**A**) and validation (**B**) cohorts

In the present study, an ANN prediction model was constructed for the first-time using machine learning and is suitable for application to individual patients. The model can evaluate the probability of EGVB within 1 year (an online version is available at https://lixuan.me/annmodel/hyx_20210320/). It combines basic patient information with laboratory markers and stratified the patients according to the estimated EGVB risk into low and high-risk groups. The ANN model performed well in predicting EGVB development at 1 year, as supported by the area under the curve (AUC) for the training and calibration curves. It showed superior predictive performance for EGVB development in liver cirrhosis patients over NIEC and Rev-NIEC index models (all *P* <  0.001). Also, decision curve analyses showed the ANN model was enhanced compared to conservative NIEC and Rev-NIEC index models Compared with the NIEC and Rev-NIEC indices, the ANN model showed net benefit improvements in both the training (Fig. 4A) and validation cohorts (Fig. 4B), which indicated that the ANN model had better clinical practicability than the other approaches.

The ANN model has the ability to “learn from each datum” and connect each input with a corresponding output, by altering the weight of neuronal connections. In comparison to logistic regression or Cox regression models, the ANN model is non-linear, and continuously directs factors related to the outcome to achieve the most suitable prediction model; thus, it has a higher predictive accuracy. Consequently, the ANN model offers unique advantages over previous risk models, including serving as a basis for EGVB screening strategies for patients with varying clinical stages of liver cirrhosis, having the ability to calculate the annual incidence of EGVB in a large sample size, and having excellent performance in both training and validation cohorts.

As expected from previous literature [11, 12, 32, 33], the sizes of varices, as well as the presence of RWM were found to be strong predictors of EGVB. Endoscopy, as the gold standard technique for varices diagnosis, has an important role to play in the assessment of the bleeding risk in patients with GEV [32]. Patients with large varices have an approximately three times higher risk for EGVB than those with small varices, while the presence of RWM increases the risk of bleeding up to four times [34, 35]. Furthermore, liver dysfunction severity, elevated GGT levels, and ascites were shown to be vital risk factors for EGVB. However, the liver function indicators were different from those employed in previous studies [11, 12], in which the Child–Pugh classification was associated with bleeding. However, the Child–Pugh scoring system includes some subjective indices (hepatic encephalopathy and ascites) and interrelated indices (serum albumin level and ascites) [17], which virtually increase the instability of the prediction in different studies. It has also been previously reported that the Child–Pugh classification is not associated with bleeding incidence [36]. Besides the HCT level was also found to be a factor affecting variceal hemorrhage occurrence since HCT is one of the most important indicator of whole blood, and decreased blood viscosity is associated with higher bleeding risk and increased bleeding severity [37] and accordingly a low HCT level has been indicated as a risk factor for variceal bleeding in previous reports [38, 39].

Our study had a number of limitations. First, it was a retrospective study, which no doubt had a degree of selection bias. Nevertheless, these results will have to be replicated in larger-scale studies, along with prospective studies. Second, the follow-up duration was 1 year, and the predictive performance of the model for long-term prognosis remains unclear. Conversely, 1 year is a reasonable time span for the development of the EGVB risk prediction model. Despite these limitations, our study provides new guidance for the selection of prevention strategies and offers an idea for developing a predictive model for EGVB in patients with GEV of other etiologies. In summary, the ANN model established in our study can be useful for estimating the first EGVB occurrence within 1 year and stratifying the bleeding risks in patients with liver cirrhosis with GEV, which can assist clinicians in determining the appropriate prophylactic strategies. However, the clinical utility and true predictive value of this nomogram need to be further verified in larger prospective studies.

## Conclusions

An ANN was used to fabricate a predictive model for the 1-year risk of liver cirrhosis patients to develop EGVB. As a risk stratification tool, the ANN model exhibited an excellent individualized prediction accuracy and might be useful in evaluating the EGVB risk in clinical practice.

## Supplementary Information

**Additional file 1: Supplementary Table 1.** Demographic and clinical characteristics of patients. **Supplementary Fig. 1.** Calibration curves of EGVB incidences for 1 year in the training (A) and validation data sets (B) of the ANN model probabilities.

## Data Availability

The datasets used or analyzed during the current study are available from the corresponding authors on reasonable request.

## Abbreviations

ALT: Alanine aminotransferase
ANN: Artificial neural networks
AUROC: Area under the receiver operating characteristic curves
CIs: Confidence intervals
C-index: Concordance index
EGVB: Esophagogastric variceal bleeding
GEV: Gastroesophageal varices
GGT: γ-glutamyl transferase
HCC: Hepatocellular carcinoma
HCT: Hematocrit
MELD: Model for end-stage liver disease
NIEC: North Italian Endoscopic Club
NLR: Neutrophil-lymphocyte ratio
RBC: Red blood cell
Rev-NIEC: revised North Italian Endoscopic Club
RWM: Red wale markings
