# Supplementary material for: Machine learning-based model for predicting the esophagogastric variceal bleeding risk in liver cirrhosis patients
Source: Diagn Pathol. 2023 Feb 23;18:29. doi: 10.1186/s13000-023-01293-0 (PMC9948468; doi:10.1186/s13000-023-01293-0)
Supplement: Supplementary file 1 — Additional file 1: Supplementary Table 1. Demographic and clinical characteristics of patients. Supplementary Fig. 1. Calibration curves of EGVB incidences for 1 year in the training (A) and validation data sets (B) of the ANN model probabilities. [file 13000_2023_1293_MOESM1_ESM.docx]

Supplementary materials

Supplementary Table 1. Demographic and clinical characteristics of patients

| **Variables** | **Total** | **Derivation cohort** | **Validation cohort** | ***P-*value**† |
| --- | --- | --- | --- | --- |
|  | (n = 1,100) | (n = 999) | (n = 101) |  |
| Age (years) | 53.0 (45.0-60.0) | 53.0 (45.0-60.0) | 53.9 ± 10.39 | 0.205 |
| Male gender | 748 (68.0) | 680 (68.1) | 68 (67.3) | 0.879 |
| Liver disease etiology |  |  |  | 0.156 |
| HBV | 714 (64.9) | 653 (65.4) | 61 (60.4) |  |
| HCV | 119 (10.8) | 112 (11.2) | 7 (6.9) |  |
| Alcoholic | 127 (11.5) | 112 (11.2) | 15 (14.9) |  |
| Others | 140 (12.7) | 112 (11.2) | 18 (17.8) |  |
| Smoking | 704 (64.0) | 643 (64.4) | 61 (60.4) | 0.878 |
| Drinking | 127 (11.5) | 127 (11.5) | 15 (14.9) | 0.234 |
| Ascites | 612 (55.6) | 559 (56.0) | 53 (52.5) | 0.502 |
| Hepatic encephalopathy | 46 (4.2) | 42 (4.2) | 4 (4.0) | 0.907 |
| Bacterial infection | 197 (17.9) | 192 (19.2) | 5 (5.0) | 0.000 |
| Location of varices at index gastroscopy |  |  |  | 0.137 |
| Gastric varices only | 43 (3.9) | 42 (4.2) | 1 (1.0) |  |
| Esophageal varices only | 438 (39.8) | 391 (39.1) | 47 (46.5) |  |
| Esophageal and gastric | 619 (56.3) | 566 (56.7) | 53 (52.5) |  |
| Size of varices |  |  |  | 0.693 |
| Small | 543 (49.4) | 491 (49.1) | 52 (51.5) |  |
| Medium | 290 (26.4) | 262 (26.2) | 28 (27.7) |  |
| Large | 267 (24.3) | 246 (24.6) | 21 (20.8) |  |
| RWM | 342 (31.1) | 307 (30.7) | 35 (34.7) | 0.417 |
| Laboratory data |  |  |  |  |
| ALT (U/L) | 36.45 (23.93-66.80) | 36.70 (24.30-66.50) | 33.60 (19.35-74.25) | 0.241 |
| AST (U/L) | 49.00 (32.80-87.08) | 50.0 (33.20-86.70) | 42.60 (27.45-92.05) | 0.139 |
| TBIL (µmol/L) | 29.10 (18.50-48.83) | 29.4 (18.7-48.3) | 25.30 (16.05-54.10) | 0.286 |
| GGT (U/L) | 51.90 (28.00-113.08) | 53.00 (28.20-113.7) | 45.30 (26.40-96.30) | 0.279 |
| ALP (U/L) | 100.75 (75.73-134.28) | 101.40 (75.80-136.00) | 96.50 (75.05-120.50) | 0.187 |
| ALB (g/L) | 31.60 (27.70-36.60) | 31.60 (27.70-36.60) | 32.11 ± 6.00 | 0.917 |
| WBC (× 10^9^/L) | 3.34 (2.59-4.63) | 3.33 (2.59-4.62) | 3.38 (2.59-4.77) | 0.901 |
| RBC (× 10^12^/L) | 3.65 (3.20-4.11) | 3.652 ± 0.711 | 3.64 ± 0.74 | 0.705 |
| PLT (× 10^9^/L) | 62.00 (45.00-87.00) | 62.00 (44.60-87.00) | 62.90 (48.25-87.5) | 0.488 |
| NLR | 1.90 (1.35-2.83) | 1.891 (1.338-2.833) | 2.00 (1.43-3.11) | 0.418 |
| HCT (%) | 35.00 (31.19-39.00) | 35.05 (31.24-39.17) | 34.50 (30.38-38.35) | 0.339 |
| K (mmol/L) | 3.69 (3.44-3.95) | 3.69 (3.45-3.95) | 3.64 ± 0.46 | 0.166 |
| NA (mmol/L) | 140.2 (137.9-142.0) | 140.20 (137.90-141.90) | 140.0 (137.3-142.5) | 0.992 |
| BUN (mmol/L) | 5.00 (3.95-6.18) | 4.98 (3.950-6.190) | 5.12 (3.99-6.185) | 0.844 |
| CREA (µmol/L) | 63.00 (55.00-74.00) | 63.40 (55.00-74.00) | 63.00 (53.45-77.00) | 0.868 |
| GLU (mmol/L) | 5.54 (4.92-6.84) | 5.52 (4.92-6.82) | 5.69 (5.08-7.20) | 0.197 |
| PT (s) | 14.70 (13.10-16.40) | 14.6 (13.1-16.4) | 14.70 (13.03-16.70) | 0.974 |
| PTA (%) | 66.00 (55.00-78.00) | 66.00 (54.90-78.00) | 66.00 (56.78-78.00) | 0.701 |
| Spleen thickness (mm) | 48.0 (42.0-55.0) | 48.00 (42.00-55.00) | 47.00 (41.00-53.50) | 0.501 |
| Portal vein diameter (mm) | 12.0 (11.0-13.0) | 12.00 (11.00-13.00) | 12.00 (11.00-13.00) | 0.560 |
| Child-Pugh grade‡ |  |  |  | 0.740 |
| A | 382 (34.7) | 346 (34.6) | 36 (35.6) |  |
| B | 536 (48.7) | 490 (49.0) | 46 (45.5) |  |
| C | 182 (16.5) | 163 (16.3) | 19 (18.8) |  |
| MELD‡ | 10 (8-13) | 10 (8-13) | 10 (8-14) | 0.832 |

Notes: Data are presented as n (%), means ± standard deviations, or medians (interquartile ranges).

^†^Comparison results between the derivation and validation cohorts

^‡^Child–Pugh grade and MELD score were not included in the least absolute shrinkage and selection operator regression analyses.

Abbreviations: ALB, albumin; ALP, alkaline phosphatase; ALT, alanine aminotransferase; AST, aspartate aminotransferase; BUN, blood urine nitrogen; CREA, creatinine; GGT, γ-glutamyl transferase; GLU, glucose; HBV, hepatitis B virus; HCT, hematocrit; HCV, hepatitis C virus; K, potassium; MELD, model for end-stage liver disease; NA, sodium; NLR, neutrophil-lymphocyte ratio; PLT, platelet; PT, prothrombin time; PTA, prothrombin time activity; RBC, red blood cell; RWM, red wale markings; TBIL, total bilirubin; WBC, white blood cell.


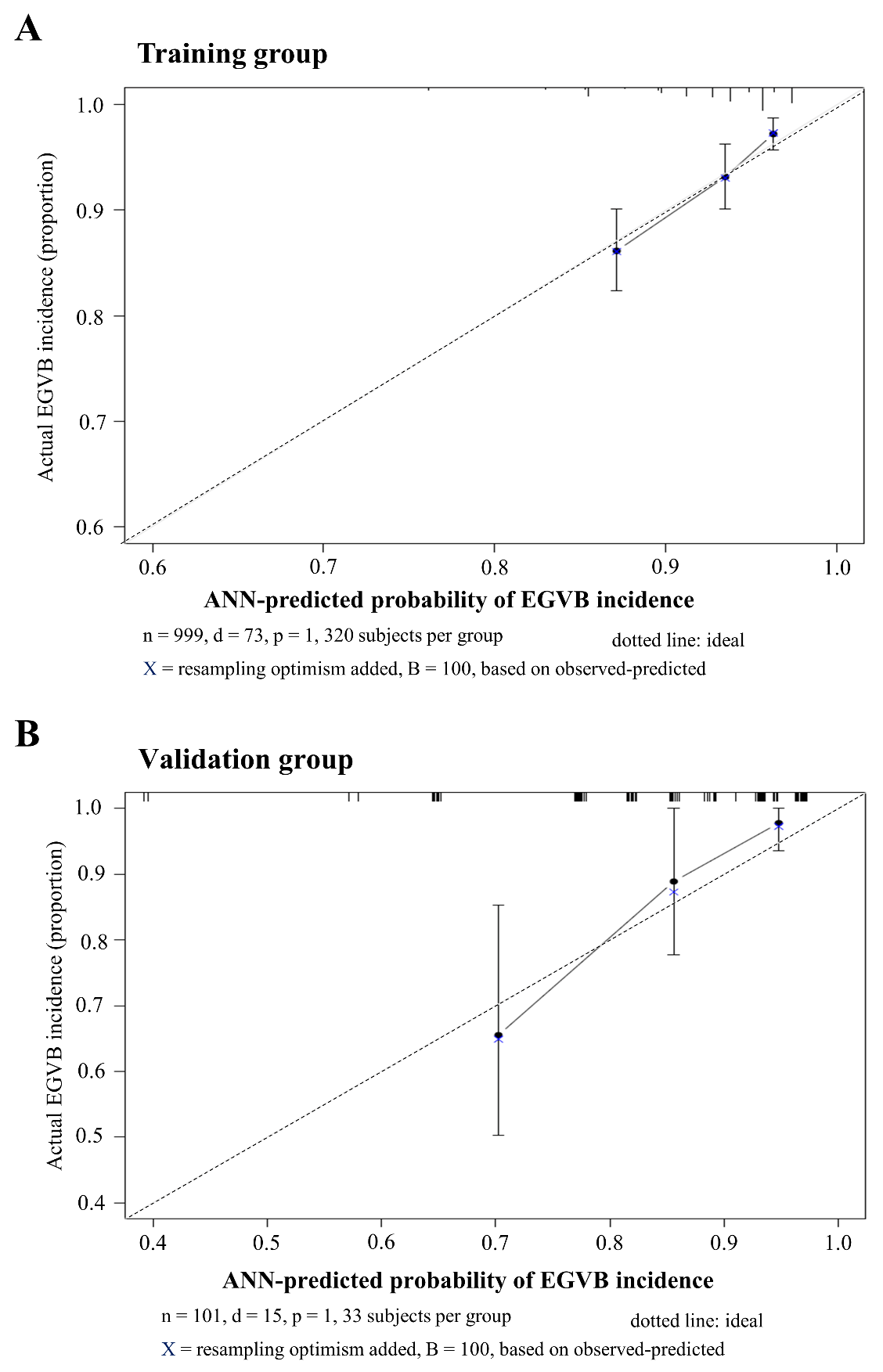


**Supplementary Figure 1.** Calibration curves of EGVB incidences for 1 year in the training (A) and validation data sets (B) of the ANN model probabilities.
